# Poly (vinyl alcohol)/β-Cyclodextrin Composite Fiber with Good Flame Retardant and Super-Smoke Suppression Properties

**DOI:** 10.3390/polym12051078

**Published:** 2020-05-08

**Authors:** Cheng-Yuan Xing, Shi-Lin Zeng, Shi-Kai Qi, Meng-Jin Jiang, Long Xu, Li Chen, Sheng Zhang, Bang-Jing Li

**Affiliations:** 1 Key Laboratory of Mountain Ecological Restoration and Bioresource Utilization, Chengdu Institute of Biology, Chinese Academy of Sciences, Chengdu 610041, China; xing919969255@163.com (C.-Y.X.); xulongcd@163.com (L.X.); 2 College of Life Sciences, University of Chinese Academy of Sciences, Beijing 100049, China; 3 State Key Laboratory of Polymer Materials Engineering, Polymer Research Institute of Sichuan University, Sichuan University, Chengdu 610065, China; zeng1s2l3@163.com (S.-L.Z.); clclover@foxmail.com (L.C.); 4 State Key Laboratory of Polymer Materials and Engineering (Sichuan University), College of Polymer Science and Engineering, Sichuan University, Chengdu 610065, China; qsk_scu@163.com (S.-K.Q.); memoggy@126.com (M.-J.J.)

**Keywords:** cyclodextrin, fiber, flame retardant, smoke suppression

## Abstract

Fibers with good flame retardant (FR) and smoke suppression performances are highly desirable for the purpose of eliminating fire hazard. This study developed a novel FR fiber by wet-spinning poly (vinyl alcohol)/β-cyclodextrin (PVA/βCD) composite fiber and crosslinking it with hexamethylene diisocyanate (HDI). βCDs showed good compatibility with PVA matrix, and the resulting PVA/CD/HDI fibers showed mechanical strength at the same level as natural cotton fiber. The PVA/CD/HDI fibers also showed excellent flame retardance (the LOI value of PVA/CD/HDI could reach 41.7%, and their peak of heat release (PHRR) could be reduced by up to 77.7% by neat PVA), and super-smoke suppression (the value of total smoke production (TSP) was only 28.6% compared to PVA). These dramatic reductions of fire hazard were ascribed to the char formation of βCD and crosslinking structure of PVA/CD/HDI, which formed a compact char layer during combustion, thus preventing heat transmission and smoke release.

## 1. Introduction

Fire accidents are harmful to life and property and cause more life and property losses than natural disasters [1]. Flammability of textile fibers, in particular, is always a severe threat for the safety of people [2]. More than 94% of fire fatalities in houses are related to burning of upholstered furniture and clothing. It has been reported that most fire casualties are due to toxic gases, oxygen deprivation, and smoke inhalation [3]. Firstly, the high temperature of smoke can cause thermal damage to people. Secondly, fire smoke contains large amounts of toxic gases, which induce poisoning and suffocation. Thirdly, the visibility reduction caused by fire smoke prevents evacuation of fire victims and interferes with rescue [4]. Therefore, reducing fire hazards requires consideration of effective smoke suppression of fibers. For example, the usage of halogen-based flame retardants (FRs) has been reduced by regulations because of the toxic and corrosive gas release during decomposition, although they endow good fire resistance to fibers [5,6]. However, it should be noted that the current widely used phosphate-containing intumescent FRs still release toxic smoke, such as phosphine, during combustion [7]. FR systems acting as both smoke suppressants and FRs are highly desired for the development of ideal flame-resistant fibers. 

In recent years, carbohydrates have been explored as a possible valuable green alternative to the conventional flame retardants for textiles since their high content of hydroxyl groups are suitable for producing thermally stable charred residues when exposed to fire [8,9,10]. A major advantage related to use of carbohydrate flame retardants is their low environmental impact and toxicity [11,12,13,14,15]. Among carbohydrates, chitosan, cellulose, and starch are widely used as carbon sources in intumescent FR systems, which can protect materials during combustion through the formation of an expanded carbonized layer [12,13,15]. It should be noted that carbohydrates usually need to cooperate with other components in order to achieve acceptable levels of fire retardance. At present, there are some limitations related to the development of efficient carbohydrate-based FR for fabric: 1) Their smoke suppression performances have not yet received attention. While smoke suppressants such as transition metal compounds and inorganic fillers should be considered, the introduction of metal compounds and inorganic particles in fibers and/or textiles faces the problems including dispersion of particles during processing, compatibility of particles with polymers during and after processing, and impairment of the spinnability fibers [16,17]; 2) The technologies to use carbohydrate-based FR at a large scale are still under evaluation. Some studies coat smoke suppressants and FR on the surface of fabrics through post-treatment, which successfully solves the process problems, although this solution suffers from poor durability.

It remains a big challenge to design ideal carbohydrate-based, flame-resistant fibers which have self-extinguishing capability after ignition, together with low and nontoxic smoke release during burning.

Cyclodextrins (CDs), carbohydrates produced from decomposition of starch, contain 18–24 hydroxyl groups (depending on the species) and are used as part of intumescent FR systems in many polymers (e.g., poly(lactic acid), poly(ethylene), poly(vinyl acetate), polyamides, and polyesters) [18,19,20,21,22]. However, little attention has been paid to the smoke suppression properties of CD, and there has been no CD-containing flame-retardant fiber reported to date. CDs have good spinnability, as demonstrated by Uyar’s group and ourselves, who have developed CD and CD composite fibers using the electrospinning method and applied them in drug delivery, antibacterial, molecular entrapment and air filtration [23,24,25,26]. Furthermore, CDs are thermally stable (i.e., the degradation temperature of βCD is up to 300 °C), which may survive from the high post treatment temperatures of the fiber [27]. It is challenging to apply CD-based FRs in fibers for two reasons. Firstly, the CD should always combine with other FRs, such as ammonium polyphosphate (APP), since CD alone is not able to enhance the combustion resistance of the materials effectively [20]. However, many FRs, such as APP will be completely degraded under the conditions of fiber post treatment. Secondly, in some cases, CD-based FR do not prevent smoke release, sometimes giving rise to higher amounts of smoke [28].

**Scheme 1:**
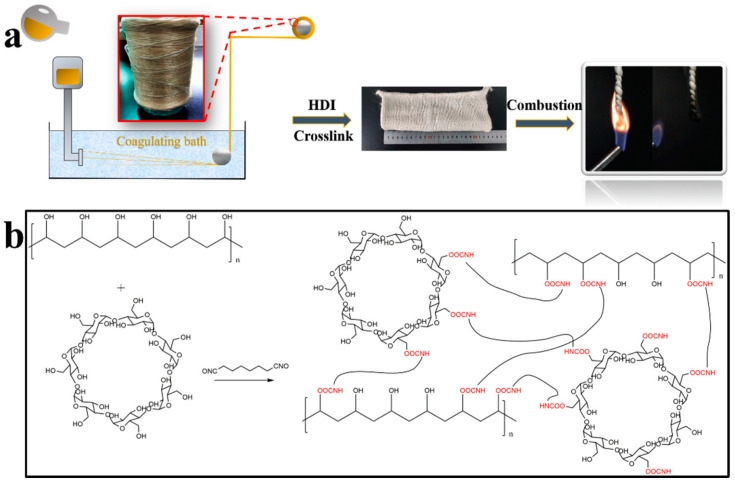
(**a**) Procedure of the total process, including the preparation of PVA or PVA/CD fibers, the crosslinking of the fibers and its combustion behavior; (**b**) Scheme of HDI crosslinking with hydroxyl group from PVA and βCD.

This study aimed to develop a kind of halogen-free and phosphonate-free FR fiber with flame retardant and smoke suppressant properties. βCD is chosen as the carbon source mixing with polyvinyl alcohol (PVA) and the PVA/βCD (PVA/CD) composite is spun to fiber by wet spinning. PVA is a water-soluble and biocompatible synthetic polymer, which shows potential in various fields such as fiber-reinforced composites, textiles, paper-making, battery separators, solid-state lighting and biomedical applications [29,30,31,32,33]. However, PVA is readily ignitable in the air. Nowadays, halogen-free flame retardants for PVA fibers are prepared mainly by blending PVA with intrinsic FR fiber, such as melamine-formaldehyde, or coating flame retardants on the fiber surface through post-treatment. For example, Xu et al. [34] prepared melamine formaldehyde/PVA composite fiber via phase separation. This method demonstrates great flame-retardant properties (LOI > 42%). However, the nitrogen loss of the fibers indicates a potential durability problem. For permanent purposes, Jiang et al. [35] introduced melamine formaldehyde (MF) to the surface of formalized PVA fiber using the grafting method. However, only a high grafting degree (>30%) results in flame-resistant fiber (LOI > 27%). Moreover, the content of MF resins leads to a sharp decrease in the mechanical properties of the fibers, indicating poor compatibility with the fiber. Thus, the problems of compatibility and durability are two of the main challenges these methods face. In this work, βCD and PVA were spun, coagulated, oriented, and crosslinked under essentially similar conditions, since both of them have abundant hydroxyl groups and have good compatibility (Scheme 1a). Wet spinning was chosen since it is more cost-effective and it is easier to realize large-scale manufacturing compared with the electrospinning method. Instead of conventional formalization of PVA, hexamethylene diisocyanate (HDI) was used to crosslink βCD and PVA to improve the water resistance of composite fiber. Furthermore, HDI acts as N source and is a synergist in the βCD-containing FR fiber. This novel FR strategy improved the flame retardance and smoke suppression of PVA dramatically.

## 2. Materials and Methods

### 2.1. Materials

PVA 1799, ammonium sulphate (NH_4_SO_4_, 95.0%) and ethyl acetate (99.5%) were purchased from Kelong Chemical Reagent Company of Chengdu (Chengdu, China). Ethyl acetate was physically dried over sodium sulphate. βCD was purchased from Tianjin Kemiou Chemical Reagent Co., Ltd (Tianjin, China). HDI was purchased from Macklin (Shanghai, China, 98.0%). Ultrapure water (UP water, resistivity > 18.0 MΩ·cm, 25 °C) was also used in this work.

### 2.2. Preparation of PVA and PVA/CD Fibers

**Figure 1:**
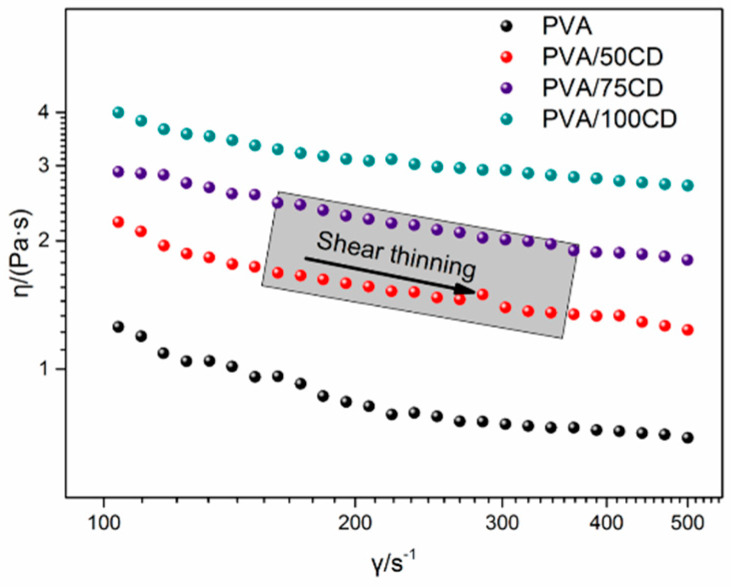
Rheological behavior of the PVA, PVA/50CD, PVA/75CD and PVA/100CD spinning dopes.

Neat PVA spinning dope was prepared by dissolving PVA in water with the concentration of 19.0% (*w*/*w*) at 95 °C. The PVA/CD dopes were prepared by dissolving PVA and βCD in water (the concentration of PVA was 19% (*w*/*w*) by mechanical stirring; the mass ratios of PVA and βCD were 1.0:0.5, 1.0:0.75, 1.0:1.0 respectively named PVA/50CD, PVA/75CD and PVA/100CD) at 95 °C to prepare spinning dopes. The obtained transparent spinning solutions were Non-Newtonian fluids (Figure 1). Wet spun fibers were prepared on a wet spinning machine made by Sichuan University kindly provided by Dr. Meng-Jin Jiang. 

The spinning dopes were extruded to a NH_4_SO_4_ coagulation bath from a spinneret with 200 nozzle holes (nozzle diameter 0.08 mm) to form the as-spun fibers. The barrel temperature was 95 °C. The temperature of the coagulation bath was 55 °C. After the primary fibers were prepared, PVA and PVA/CD fibers were hot stretched and hot set, respectively. The hot stretch ratio was 1:2 and the hot set temperature was 180 °C.

### 2.3. Preparation of Crosslinked PVA and PVA/CD Fibers

The PVA and PVA/CD fibers were crosslinked by HDI as follows: 5.0 g fibers were immersed in 100 mL ethyl acetate solution containing 2.5% (*v*/*v*) HDI. Ethyl acetate was physical dried before use. The detailed process was as follows: 50.0 g anhydrous sodium sulphate was added to 500 mL ethyl acetate for 12 h to remove water from ethyl acetate. The fibers were crosslinked in stretching state. The crosslinking was put forward at 77 °C for 24 h [36]. Crosslinked fibers were washed by water, ethanol and water consecutively until no turbid could be observed. The crosslinked fibers were finally dried in a drying box at 50 °C for 5 h.

### 2.4. Characterization

The rheological properties and spinnability of PVA and PVA/CD dopes were investigated on a cone rheometer Gemini 200 (Malvern Instruments, Malvern, UK), cone-plate diameter 30 mm and cone angle 1°. Rheological measurements were carried out at 95 °C. The surface morphology of the fibers was studied using a Scanning Electron Microscope (SEM) (JSM-7500F, JEOL, Tokyo, Japan), whose beam voltage was 15 kV, then fixed with conductive adhesive tapes and coated with gold. The fibers were then cut off with a new blade in the bound state to study the morphology of cross section. The diameter distribution was conducted by ten similar cross section (the long side) images from SEM. The measurements of 100 fibers were used in order to obtain a statistically meaningful result. Fourier transform infrared spectrometer (FTIR) (Nexus-560, Nicolet, Madison, WI, USA) was used to confirm the crosslinked characteristic peak. The fibers were cut into powder form before testing. The scan range was from 400 cm^−1^ to 4000 cm^−1^ with a resolution of 4 cm^−1^. The mechanical properties of fibers were measured by an electronic single fiber strength tester (YG001A, HDFY, Shanghai, China). A minimum of 10 fibers were tested and averaged for final results. The drawing speed was 20 mm/min. The linear density was calculated based on 100 fibers and initial length was 100 mm. X-ray diffraction (XRD) measurement was carried out on a Bruker D8 Advance X-ray diffractometer (Bruker, Karlsruhe, Germany) using Cu Kα radiation in the scattering angle range of 2θ = 5 ~ 30° at a scan speed of 4° min^−1^. The thermal stability of the fibers was evaluated by thermogravimetric analysis (TGA) (Q600, TA Instruments, New Castle, DE, USA) in air atmosphere, the fibers were heated to 600 °C with a heating rate of 10 °C min^−1^. A TA instrument Q600 analyzer was used to analysis the samples, 5 mg fiber powder was placed in open alumina pans under air atmosphere. Before flame retardancy testing, PVA and PVA/CD fibers were washed in water/ethanol mixtures to remove NH_4_SO_4_ from the coagulation bath. Flame testing was carried out by a horizontal and vertical burning instrument (GZF-5, Wuhan Gramo Testing Equipment Co., Ltd., Wuhan, China). The vertical flame tests aimed to realize a UL-94 ranking according to the procedure described in the ASTM D4804 standard (Sample dimensions 12.5 mm × 10.0 mm × 8 mm). Limiting Oxygen Index (LOI) tests were carried out by a JF-3 Oxygen Index Measuring Instrument (Jiangsu Zhenguitaibang electronic technology Co. Ltd., Nanjing, China) according to the ASTM D2863 standard. Cone calorimetry tests (FTT0007, Fire Testing Technology, East Grinstead, UK) were conducted according to the ISO 5660 standard, the size of the samples were 100 × 100 × 4 mm by hot pressing. The samples were irradiated at a heat flux of 50 kW·m^−2^ in a horizontal configuration. The residues after cone calorimeter testing were evaluated by SEM and Raman spectroscopy (DXRxi, Thermal Fischer, Waltham, MA, USA). The Raman spectra was recorded from 900 to 2000 cm^−1^ using a 785 nm argon ion laser. 

## 3. Results

### 3.1. Preparation of PVA/CD/HDI Fibers

The compatibility between PVA and βCD solution is good because all the PVA/CD solutions were optically clear. Their rheological properties were investigated since they have an important influence on the spinnability and physical properties of composite fibers. Figure 1 is the relationship between shear rate and viscosity of spinning solutions at 95 °C. PVA solution is a typical non-Newtonian fluid showing shear thinning. The addition of βCD does not change the rheological properties of the PVA spin dopes obviously. All of the PVA/CD spin dopes show good solution stability at high shear rate and their viscosities are acceptable for spinning purposes [37]. The NH_4_SO_4_ coagulation bath coagulates PVA and PVA/CD composite filaments very well. The nascent fibers were hot-stretched and hot-set to be oriented. The mass ratios of PVA and βCD in PVA/CD are 1.0:0.5, 1:0.75, 1.0:1.0 respectively, and the obtained fibers are denoted as PVA/50CD, PVA/75CD, and PVA/100CD. The PVA and PVA/CD fibers were crosslinked by HDI to improve the water-resistance and flame retardance of fibers. The elemental analysis (EA) results of PVA/HDI and PVA/75CD/HDI are listed in Table S1. It was found that the both fibers contained nitrogen after crosslinking. PVA/75CD/HDI had greater N and O contents, suggesting higher crosslinking density after addition of βCD.

**Figure 2:**
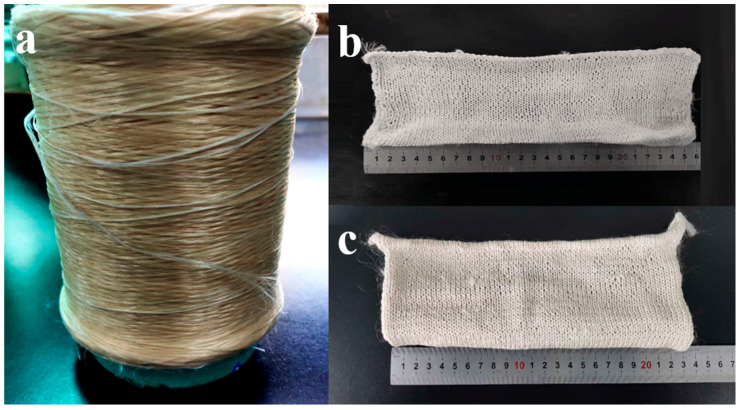
Optical images of PVA (**a**), PVA/75CD (**b**), and PVA/75CD/HDI (**c**) fabric; surface.

**Figure 3:**
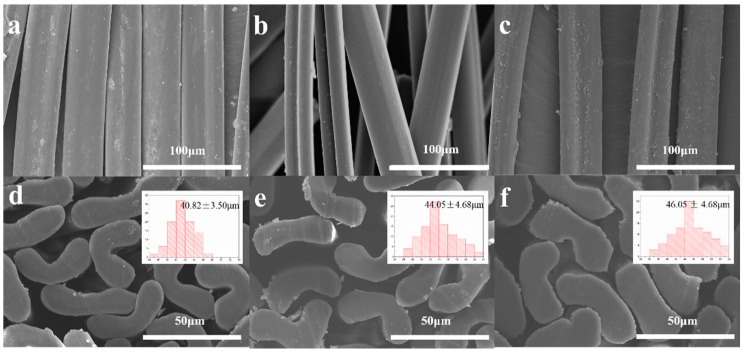
Cross sections of PVA fibers (**a**,**d**); surface and cross sections of PVA/75CD fibers (**b**,**e**); surface and cross sections of PVA/75CD/HDI fibers (**c**,**f**).

The PVA, PVA/75CD and PVA/75CD/HDI fibers show a similar morphology and can be weaved into fabric (Figure 2). The diameters of PVA, PVA/75CD and PVA/75CD/HDI fibers are 40.82 ± 3.50, 44.05 ± 4.68 and 46.05 ± 4.68 μm, respectively (Figure 3d–f). The addition of βCD increased the diameter of fiber slightly, but crosslinking does not show any obvious influence. A kidney-shaped cross-sectional structure (Figure 3 and Figure S1) is exhibited by all the fibers. The formation of kidney-shaped fiber is related to the coagulation process. Briefly, (a) the surface of the polymer is solidified during the first interaction with coagulation bath; (b) the inner solvents are diffused to the surface drawn by osmotic pressure difference; (c) the cortex is depressed for the internal vacancy and formed kidney shape [38].

**Figure 4:**
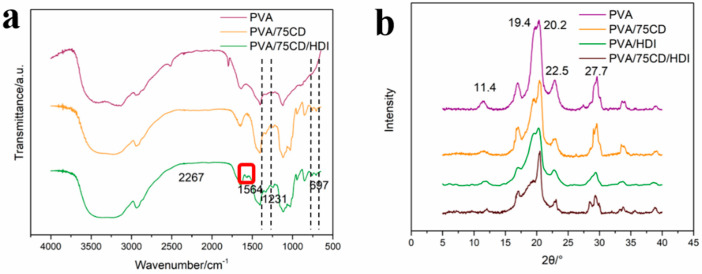
(**a**) FT-IR of as-prepared PVA, PVA/75CD and PVA/75CD/HDI fibers; (**b**) XRD of as-prepared PVA, PVA/75CD and PVA/75CD/HDI fibers.

It has been reported that the hydroxyl group in βCD and PVA can react with isocyanate (—NCO) very well (Scheme 1b) [36,39]. Figure 4a shows the FT-IR spectra of PVA, PVA/75CD and PVA/75CD/HDI fibers. Compared with PVA and PVA/75CD, PVA/75CD/HDI shows new absorption at 1555 cm^−1^ assigned to a NH—CO bending vibration [40], indicating successful crosslinking. In addition, the characteristic signal at 2267 cm^−1^ from —NCO disappears, implying that the —NCO groups have been consumed during crosslinking and washing. Figure 4b shows the XRD patterns of PVA, PVA/HDI, PVA/75CD and PVA/75CD/HDI. It can be seen that the addition of βCD and crosslinking both result in the reduction of crystal peak intensity, implying that the decrease of crystallinity.

### 3.2. Mechanical Properties of PVA/CD/HDI Fibers

As shown in Table 1, the introduction of βCD and HDI results in the decrease of breaking strength and increase of elongation at break, which may be caused by the decrease of crystallinity (Figure 4b). It should be noted that the breaking strength of the PVA/CD/HDI fibers are still at the same level as natural cotton fiber [41]. The PVA/CD/HDI fibers show higher linear density than PVA/CD and PVA, since the density of βCD (1.6 g/cm^3^) is greater than that of PVA (1.2 g/cm^3^) and crosslinking further increases the density.

### 3.3. Flame Retardant Assessment

**Figure 5:**
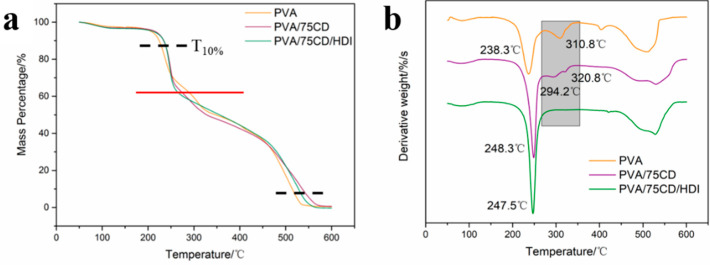
(**a**) TGA curves of PVA, PVA/75CD and PVA/75CD/HDI fibers under air atmosphere; (**b**) DTG curves of PVA, PVA/75CD and PVA/75CD/HDI fibers under air atmosphere

TGA was conducted in an air atmosphere to evaluate the thermal oxidative degradation behavior of the three fibers [42]. As shown in Figure 5a, the weight loss of the fibers undergoes three stages: side group dehydration, main chain degradation, and char degradation [43,44]. The T_10%_ (the temperature at 10% material loss, Figure 5a and Table S2) of the crosslinked sample (PVA/75CD/HDI) was a little higher than that of the uncrosslinked samples (PVA, PVA/75CD). At around 550 °C under air atmosphere, PVA/75CD and PVA/75CD/HDI showed higher residue weight percentages (W_550 °C_, 5.5% and 6.4%, Table S2) than the control PVA fiber (0.9%). These results indicate that the thermal stability of fiber is improved by the introduction of βCD and crosslinking. The thermal stability of the three fibers was also assessed by derivative thermogravimetry (DTG) (Figure 5b). From DTG, we can see that the peak weight loss increased from 238.3 °C to 248.3 °C with the addition of βCD, indicating strengthened thermal stability. It should also be noted that peak weight loss at 310.8 °C (the gray area) disappeared for PVA/75CD/HDI. It seems that the introduction of HDI slows down the further decomposition of PVA.

LOI and UL94 tests were performed to investigate the flame retardance of the fibers, and the results are shown in Table 2. It can be seen that the addition of βCD improves the flame retardance of the fibers, but the LOI value of PVA/CD is still below 27.0% and the fiber still could be ignited even when the amount of βCD was equal to PVA, suggesting that the FR efficiency of the single component of βCD was not enough. In this work, the combination of βCD and crosslinker HDI increases the FR efficiency significantly. It is shown that the LOI value of PVA/75CD/HDI reaches 41.7%, which is much higher than the single component flame-retardant PVA composite fibers (PVA/75CD and PVA/HDI) and meets the requirement of non-combustible (LOI > 32.0%) fiber. Table 2 shows the UL94 results of PVA, PVA/HDI, PVA/75CD/HDI fibers. It can be seen that the PVA/75CD/HDI fibers showed the best results. Both PVA/75CD/HDI and PVA/HDI fibers reached V0 level. However, it should be noticed that the afterflame times of PVA/75CD/HDI are zero, indicating the better flame resistance.

**Figure 6:**
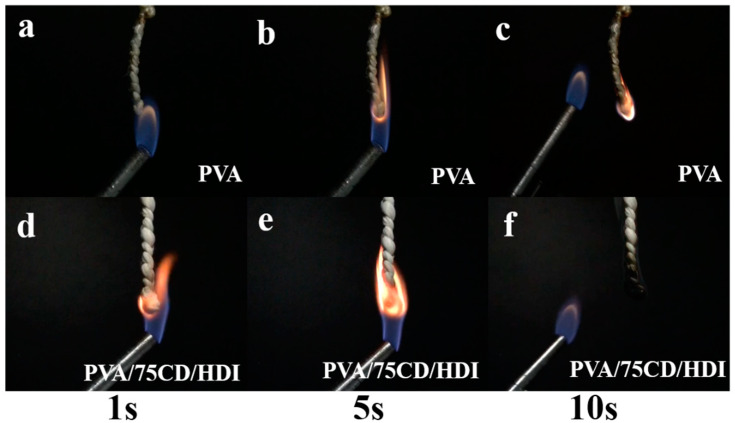
Fire ignition behavior for as-prepared combustible PVA fiber: (**a**) before ignition; (**b**) being ignited; and (**c**) combustion behavior of the fiber after ignition. Fire ignition behavior for flame-retardant PVA/75CD/HDI fiber: (**d**) before ignition; (**e**) being ignited; and (**f**) fire status of the fiber after ignition.

Figure 6 shows the combustion behavior of PVA and PVA/75CD/HDI in air. It can be seen that the neat PVA fiber was continuously consumed after ignition, while the PVA/75CD/HDI fiber extinguished right after ignition (Videos S1 and S2). These results also demonstrate good synergistic effect of βCD and HDI.

**Figure 7:**
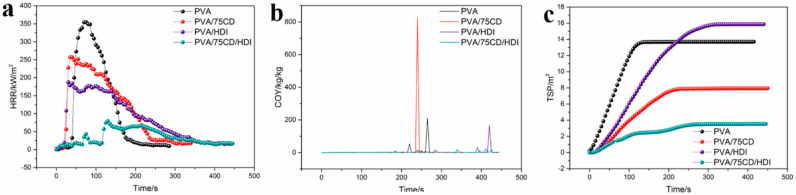
Cone calorimetry results of (**a**) heat release rate and (**b**) CO yield (COY) of PVA, PVA/75CD, PVA/HDI and PVA/75CD/HDI fibers; (**c**) TSP of PVA, PVA/75CD, PVA/HDI and PVA/75CD/HDI fibers.

For cone calorimetric studies, the selected source power, 50 kW/m^2^ was chosen to simulate the flame in the middle of the fire [45]. The corresponding parameters, including time to ignition (TTI), peak of heat release (PHRR), total heat release (THR), average specific extinction area (avSEA), average mass loss rate (avMLR), char residue, total smoke release (TSR) and total smoke production (TSP) were obtained to evaluate flammability and toxicity of the fibers. The results are shown in Table 3. As shown in Figure 7a, the presence of βCD alone decreased the PHRR value to a certain extent compared with control PVA fibers (27.6% decrease for PVA/75CD); however, time to ignition did not increase. In the case of PVA/75CD/HDI, it showed not only significant PHRR decrease (77.7% decrease compared with PVA fibers), but also longer ignition time. The TTI increased from 35 s to 66 s. After ignition, the combustion of PVA/75CD/HDI produced a smaller amount of heat, until 120 s, when it began to sustain a very low level of heat release. Compared with PVA fibers, which showed a significant heat release after ignition, PHRR of the PVA/75CD/HDI decreased 77.7%. PHRR reduced maximum temperature and flame spread rate [46], which means the fire hazard of PVA/75CD/HDI was greatly decreased.

It is interesting that the THR values of PVA, PVA/75CD and PVA/HDI were 33, 38, and 38 MJ/kg, respectively, while the value of PVA/75CD/HDI was only 16 MJ/kg, exhibiting a significant reduction. These results indicate that the combination of βCD and HDI show great flame retardancy of PVA. In the combustion process, PVA/75CD/HDI prevents the emission of most of the heat, which could inhibit the spread of the flame to some extent.

Figure 7b presents the curves of the CO production for PVA, PVA/75CD, PVA/HDI and PVA/75CD/HDI fibers. It can be seen that the introduction of HDI can reduce the CO release, and the CO release was reduced further by combination of HDI and βCD. The maximum intensity of the CO in PVA/75CD/HDI was reduced by 88.45% compared to neat PVA fibers. At the same time, the maximum intensity of the CO_2_ in PVA/75CD/HDI also was reduced by 89.45% (Figure S2). These results imply that the smoke suppression of PVA/75CD/HDI is mainly attributable to the reduction of CO and CO_2_ release.

Figure 7c shows the smoke suppression performance of the PVA, PVA/75CD, PVA/HDI and PVA/75CD/HDI fibers. In the presence of βCD alone, the TSP value decreased from 14 m^2^ of neat PVA to 8 m^2^ in PVA/75CD and the smoke release rate also decreased. In the presence of HDI alone, the smoke release rate decreased slightly, while the TSP value increased. However, the combination of βCD and HDI decreased smoke release by more than 70%. The value of TSP in PVA/75CD/HDI decreased to 4 m^2^. The PVA/75CD/HDI shows good smoke suppression ability.

### 3.4. Flame Retardant Mechanism

**Figure 8:**
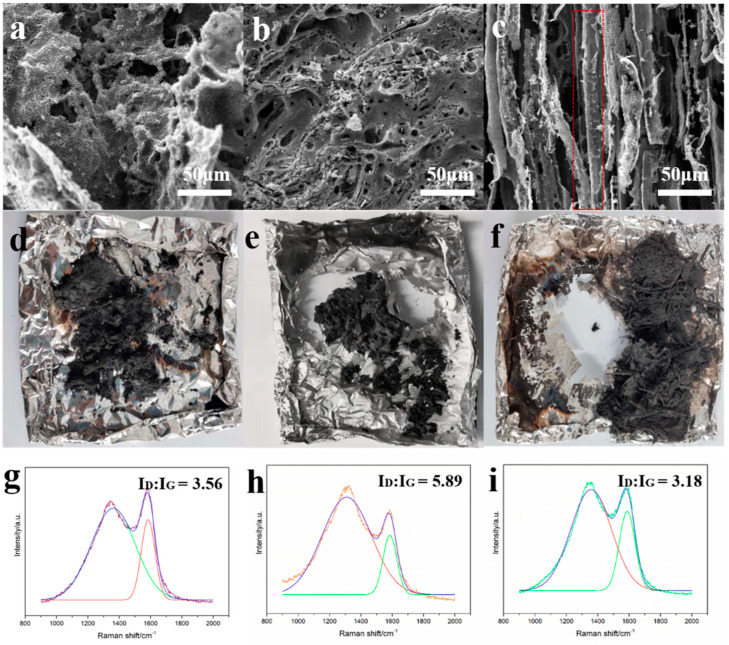
SEM images of PVA (**a**); PVA/75CD (**b**) and PVA/75CD/HDI (**c**) fibers after cone calorimetry test; Optical images of PVA (**d**); PVA/75CD (**e**) and PVA/75CD/HDI (**f**) fibers after cone calorimetry test; Raman spectra of PVA (**g**), PVA/75CD (**h**), and PVA/75CD/HDI (**i**) fibers after cone calorimetry test.

To understand the flame retardance and smoke suppression mechanism of PVA/75CD/HDI fiber, the char residues collected after cone calorimetry testing of different samples were investigated by SEM and Raman spectroscopy. As shown in Table 3, the char residue percentages of PVA, PVA/75CD, PVA/HDI and PVA/75CD/HDI fibers were 1.78%, 3.85%, 2.38%, and 13.39%, respectively. The addition of βCD is beneficial for char formation, as has also been reported by other works in the literature [20,21,22,47]. However, the surface of carbon residues of PVA/75CD fiber was quite coarse and was distributed with many pores (Figure 8b). This porous char layer cannot insulate the heat and volatile gases effectively. In contrast, the char residue of PVA/75CD/HDI fiber was not only much higher than that of PVA/75CD, but also more compact. As shown in Figure 8c, the char of PVA/75CD/HDI fiber almost had no pores and compact. Most fibers still kept their morphology. In other words, the char can be seemed to appear as a char “coating” on the surface of fibers. This can also be seen in Figure 8f (char residue after cone calorimetry testing). However, no fiber-shaped char residues can be found in Figure 8d,e. Only a concentration of char residue and some fragments can be distinguished.

**Figure 9:**
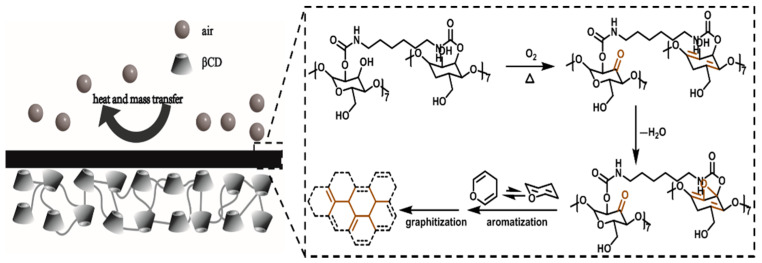
Schematic of the mechanism of condensed phase flame retardant, including oxidation, dehydration, aromatization and graphitization to form stable, compact and protective char layer.

A possible mechanism of charring for PVA/75CD and PVA/75CD/HDI fibers during the combustion can be proposed. In the case of PVA/75CD, the βCD decomposed to char through a loss of the glucosidic structure and hydroxyl groups and buildup of unsaturation, carbonyl groups and aromatic structure [20]. The high content of hydroxyl groups, βCD, acts as a C resource, thereby enhancing char formation. The introduction of HDI slows down the decomposition of PVA and strengthens the char residue (Figure 8g–i) [48]. In the case of PVA/75CD/HDI, the βCD is crosslinked with the PVA matrix by HDI, as shown in Scheme 1b and Figure 9. During the combustion, the crosslinking PVA/75CD/HDI structure was firstly oxidized in the presence of O_2_ to produce the carbonyl, ether and unsaturated double bonds (C=C). Subsequently, further crosslinking reaction occurs due to the intramolecular and intermolecular dehydration [20,28,44]. The crosslinked structure leads to formation of compact char residues. This compact char layer now acts as a barrier, which can effectively limit heat transfer, thus protecting the underlying substrate from further thermal decomposition during combustion (Figure 9). Furthermore, the condensed char layer can also effectively prevent smoke release.

## 4. Conclusions

We have successfully developed a novel flame-retardant fiber with significant smoke suppression properties by wet-spinning PVA/βCD composites and crosslinking them with HDI. The PVA/CD/HDI fibers show mechanical strengths at the same level as natural cotton fiber, and excellent flame retardancy (e.g., LOI values of PVA/75CD/HDI fiber reach 41.7%). The polyhydroxyl structure of βCD contributes to char formation. Combined with HDI, βCD formed a compact char layer and reduced smoke release to a large degree. The compact char layer acts as a dense barrier, resulting in a significantly better combustion resistance than other FR PVA fibers. The PHRR and TSP values of PVA/75CD/HDI fiber are decreased by 77.7% and 71.4%, respectively, compared with neat PVA. Such reductions in heat and smoke release can reduce the hazards of flame spread and smoke greatly. Furthermore, βCD showed good compatibility with the PVA matrix, and the PVA/CD/HDI can be prepared by facile wet-spinning and post-treatment. This potentially cost-effective and facile preparation makes it easy to realize large-scale manufacture.

## Figures and Tables

**Table 1:** Linear density and mechanical properties of uncrosslinked and crosslinked fibers.

Sample	Linear Density/dTex	Breaking Strength/cN/dTex	Elongation at Break/%	Young’s Modulus/cN/dTex
PVA	4.40	3.29 ± 0.23	30.45 ± 4.37	80.68 ± 8.50
PVA/50CD	6.70	2.12 ± 0.13	34.15 ± 5.14	59.12 ± 6.61
PVA/75CD	6.90	2.01 ± 0.35	36.70 ± 6.70	55.01 ± 6.34
PVA/100CD	7.20	1.89 ± 0.14	38.76 ± 5.19	47.80 ± 6.54
PVA/HDI	5.40	2.66 ± 0.26	47.74 ± 5.10	65.77 ± 10.37
PVA/50CD/HDI	7.50	1.93 ± 0.22	56.22 ± 7.26	45.51 ± 9.29
PVA/75CD/HDI	7.80	1.60 ± 0.10	58.36 ± 7.71	39.66 ± 4.40
PVA/100CD/HDI	8.10	1.45 ± 0.18	60.49 ± 10.03	37.31 ± 5.19

**Table 2:** LOI and UL94 ranking of PVA, PVA/HDI, PVA/75CD/HDI fibers

Sample	LOI/%	UL94	T1 ^a^/s	T2 ^b^/s	Dripping	Ignite Cotton
PVA	18.8 ± 0.2	Failed	-	-	No	No
PVA/75CD	21.9 ± 0.3	V1	10.6 ± 3.4	45.8 ± 15.9	No	No
PVA/HDI	27.9 ± 0.3	V0	0.4 ± 0.9	2.8± 1.9	No	No
PVA/75CD/HDI	41.7 ± 0.4	V0	0 ± 0	0 ± 0	No	No

^a^ Afterflame time registered in the first flame application; ^b^ Afterflame time registered in the second flame application.

**Table 3:** Cone calorimeter statistical data of PVA, PVA/75CD, PVA/HDI and PVA/75CD/HDI fibers.

Sample	PVA	PVA/75CD	PVA/HDI	PVA/75CD/HDI
TTI/s	35	19	17	66
PHRR/kW/m^2^	355	257	187	79
THR/MJ/kg	33	38	38	16
*av*SEA/m^2^/kg	704	373	676	188
*av*MLR/mg/s	65	62	53	35
TSR/m^2^/m^2^	1550	898	1795	399
TSP/m^2^	14	8	16	4
Char residue/%	1.78	3.85	2.38	13.40

## Supplementary Materials

The following are available online at https://www.mdpi.com/2073-4360/12/5/1078/s1: Figure S1. Surface and cross section of PVA/HDI, together with its diameter distribution. Figure S2. CO_2_ yield (CO_2_Y) of PVA, PVA/75CD, PVA/HDI and PVA/75CD/HDI in cone calorimeter. Table S1. Elemental analysis (EA) of PVA/HDI and PVA/CD/HDI. Table S2. Characteristic joints of TGA at air atmosphere. Video S1: Flammability of PVA, Video S2: Flammability of PVA75CDHDI.
