# Supplementary material for: Poly (vinyl alcohol)/β-Cyclodextrin Composite Fiber with Good Flame Retardant and Super-Smoke Suppression Properties
Source: Polymers (Basel). 2020 May 8;12(5):1078. doi: 10.3390/polym12051078 (PMC7284589; doi:10.3390/polym12051078)
Supplement: Supplementary file 1 [file polymers-12-01078-s001.pdf]

Supporting Information

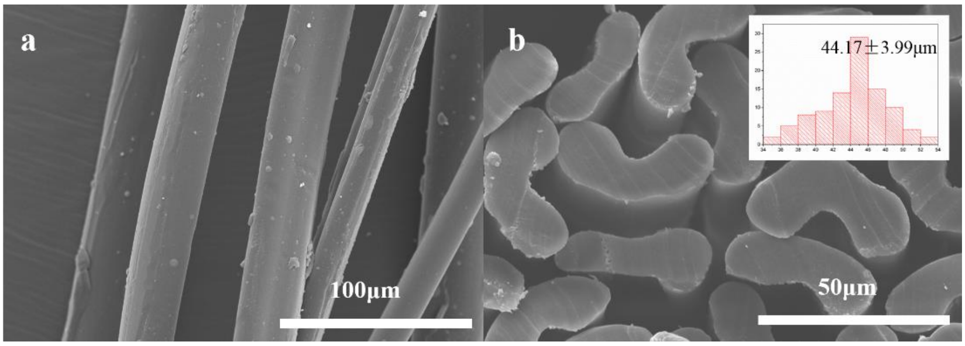

Figure. S1 Surface and cross section of PVA/HDI, together with its diameter distribution.

Table S1 Elemental analysis (EA) of PVA/HDI and PVA/CD/HDI.

Sample	C/%	H/%	O/%	N/%
PVA/HDI	51.21	9.22	39.04	0.53
PVA/75CD/HDI	45.84	8.47	42.82	2.87

Table S2 Characteristic joints of TGA at air atmosphere.

Sample	$T_{10\%}$/℃	$T_{peak\ loss}$/℃	$T_{90\%}$/℃	$W_{550℃}$/%
PVA	223.3	238.3	514.2	0.9
PVA/75CD	233.3	248.3	535.8	5.5
PVA/75CD/HDI	234.2	247.5	527.5	6.4

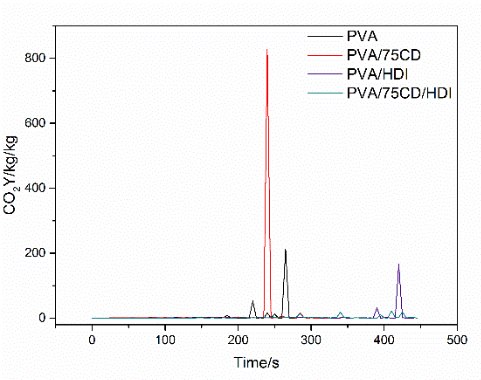

Figure. S2 CO_2 yield (CO_2Y) of PVA, PVA/75CD, PVA/HDI and PVA/75CD/HDI in cone calorimeter.
